# Corrective Bracing for Severe Idiopathic Scoliosis in Adolescence: Influence of Brace on Trunk Morphology

**DOI:** 10.1100/2012/435158

**Published:** 2012-04-30

**Authors:** Edyta Kinel, Tomasz Kotwicki, Wanda Stryła, Andrzej Szulc

**Affiliations:** ^1^Department of Rehabilitation, University of Medical Sciences of Poznan, Ul. 28 Czerwca 1956r. Nr. 135/147, 61-545 Poznan, Poland; ^2^Department of Pediatric Orthopedics and Traumatology, University of Medical Sciences of Poznan, Ul. 28 Czerwca 1956r. Nr. 135/147, 61-545 Poznan, Poland

## Abstract

*Aim*. The aim of the work was to study whether wearing a corrective brace by adolescent girls with severe idiopathic scoliosis can influence external shape of the trunk. *Methods*. Comparison of clinical deformity of two groups of girls matched for age and Cobb angle: group (1) of 23 girls, aged 14.9 ± 1.3 years, Cobb angle 55.0° ± 6.8°, who refused surgical treatment and have been wearing Chêneau brace for more than 6 months, compared with group (2) of 22 girls, aged 14.1 ± 1.8 years, Cobb angle 59.7° ± 14.6° never treated with corrective bracing. Clinical deformity was assessed with the Bunnell scoliometer (angle of trunk rotation ATR) and surface topography (posterior trunk symmetry index POTSI and Hump Sum HS). *Results.* The ATR in the primary curvature was 11.9° ± 3.4° (5°–18°) in group 1 versus 15.1° ± 5.6° (6°–25°) in group 2 (*P* = 0.027). The HS was 16.8° ± 3.8 versus 19.2° ± 4.6, respectively, *P* = 0.07. The POTSI value did not differ between groups. *Conclusion*. Girls with Cobb angle above 45 degrees, who have been subjected to brace treatment, revealed smaller clinical deformity of their back comparing to nontreated girls having similar radiological curvatures.

## 1. Introduction

According to the generally accepted indications for conservative management of idiopathic scoliosis, the brace treatment is considered a standard management for progressive curvatures of moderate Cobb angle; it is usually recommended for angles of 25 to 40 degrees and if residual growth of the spine is expected [1]. Patients with scoliosis over 45 degrees are usually advised to undergo surgical correction. In our clinical practice, we have been confronted to situations that the brace was wearing by patients with Cobb angle above 45 degrees, due to their decisions motivated with a desire of avoiding surgery. We decided to evaluate the clinical and radiological data of this group of patients consisting of 23 girls who refused surgical treatment but stayed at our observation.

Cobb angle is considered the gold standard to evaluate the curve magnitude on radiographic examination [2, 3]. The main clinical parameters in assessing trunk morphology are the C7 plumb line, shoulder and hip asymmetry which can be objectively measured with surface topography using the posterior trunk symmetry index (POTSI index) [4] and the trunk rotation measured with surface topography by calculating the Hump Sum parameter as well as the angle of trunk rotation which is measured with the scoliometer [5].

**Figure 1:**
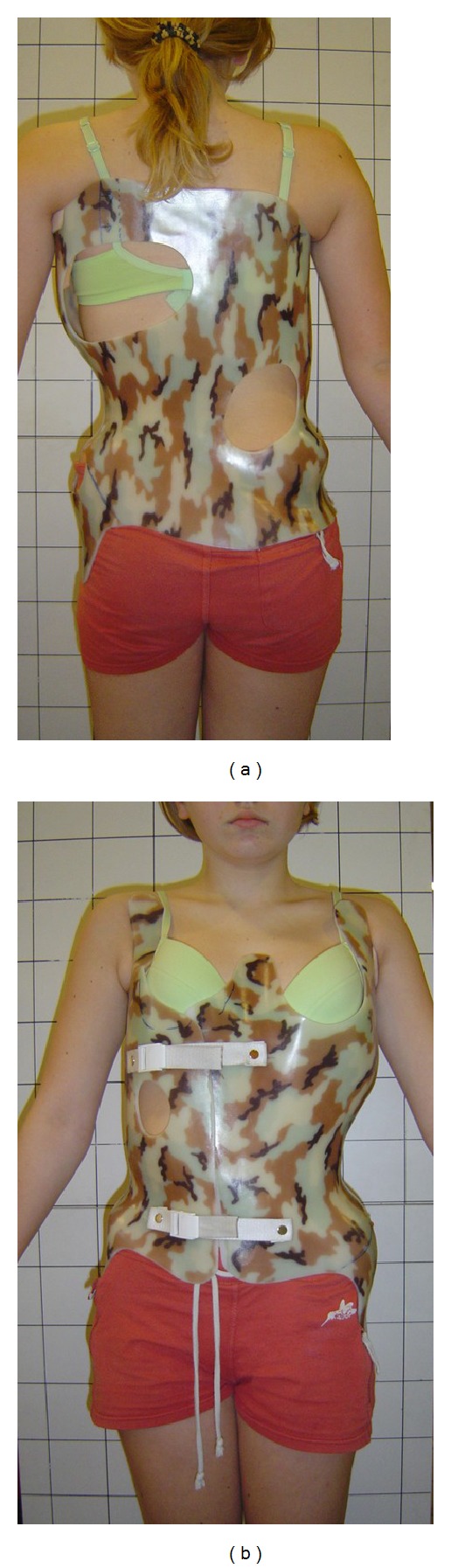
Anterior and posterior view of a patient wearing the Chêneau brace.

The aim of the study was to compare the clinical deformity in two groups of adolescent idiopathic scoliosis girls presenting similar radiological deformity: the first treated with a corrective brace (Figure 1) and the second admitted for surgical treatment.

**Figure 2:**
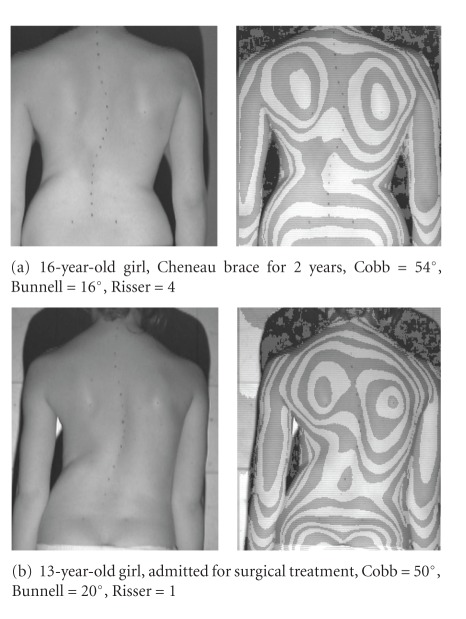
Two girls with right thoracic scoliosis of similar apex level. Digital photo (left) and surface topography (right) of the back. The surface topography image is presented in pseudo-Moire form for convenience.

The hypothesis was that girls treated for idiopathic scoliosis with a brace for a period longer than 1 year, having the curves above than 45 degrees of Cobb angle, may present significantly different morphology of the trunk comparing to girls matched for age and Cobb angle but never treated with bracing (Figure 2).

## 2. Patients and Methods

Inclusion criteria were as follows: girls, idiopathic scoliosis, Cobb angle above 45°. Forty-five patients were included in the study and distributed into two groups. The braced group consisted of 23 consecutive girls wearing a TLSO (Chêneau brace) [6], who refused surgical treatment and who have been previously wearing a TLSO brace (Chêneau) for more than 6 months. The treatment time with corrective brace varied from 8 months to 3.5 years. The patients were wearing the brace full time, (for 20 hours per day or more). The patients wearing the brace were ordered home physiotherapy with half an hour per day intensity completed with checkouts by a specialized physiotherapist at control visits. Group (2) admitted for surgical treatment consisted of 22 consecutive girls. The age of braced group was 14.9 ± 1.3 years, and the age of group admitted for surgical treatment was 14.1 ± 1.8 years, difference not significant (unpaired *t* test, *P* = 0.14).

**Figure 3:**
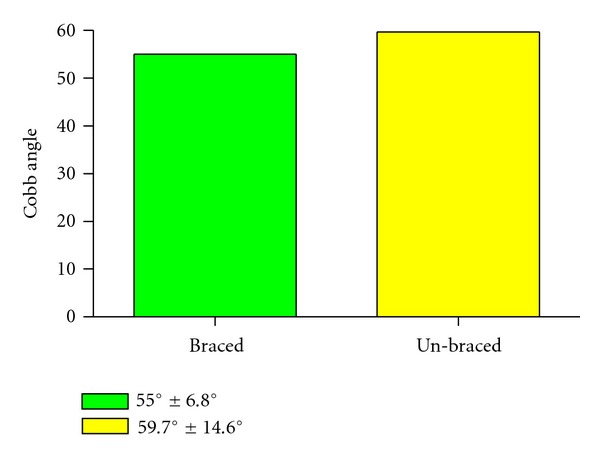
Cobb angle value in the brace-treated and admitted for surgical treatment group, difference not significant, *P* = 0.67.

The Cobb angle was 55.0° ± 6.8° (from 45° to 68°, median 55°) and 59.7° ± 14.6° (from 45° to 86°, median 54°), respectively, difference not significant (unpaired *t*-test, *P* = 0.67), (Figure 3).

Risser sign value was less than 3 in 7 girls from the braced group and in 18 girls admitted for surgical treatment. The curve pattern was similar in both groups (Table 1). In the braced group (*n* = 23), there was 15 girls with single curvatures (13 thoracic and 2 thoracolumbar) and 8 girls with double curvatures (right thoracic and left lumbar). In the group admitted for surgical treatment (*n* = 22), there was 15 girls with single curvatures (13 thoracic and 2 thoracolumbar) and 7 girls with double curvatures (right thoracic and left lumbar).

The braces were all made in the same workshop, and the treatment was managed by the same physician.

The Cobb angle out of brace was measured on standard standing frontal radiographs. The angle of trunk rotation (ATR or Bunnell angle) was measured with the scoliometer of Bunnell [5]. The ATR was measured at three levels of the spine: proximal thoracic (Th1–Th5), main thoracic (Th5–Th12), lumbar or thoracolumbar (Th12-L4), and the sum of three ATRs was calculated. All scoliometer measurements were done by the same observer (T. Kotwicki), who previously checked the rates for interobserver and intraobserver reliability and obtained a high intra-observer agreement [7].

The following clinical parameters were also considered: C7 plumb line, left and right axillary plumb line symmetry. Surface topography examination was performed the same day as the clinical and radiological examination. Raster stereography was used (CQ Electronic, Poland). The POTSI index was calculated for the frontal plane assessment and the Hump Sum (HS) for the transverse plane assessment. The HS was composed of maximum rotation at three levels of the spine (proximal thoracic, main thoracic, and thoracolumbar or lumbar).

The Kolmogorov-Smirnof test was used to check normality and the Fisher-Snedecor test to check equality of standard deviations between groups. Unpaired *t*-test was used to compare means, the Pearson coefficient for correlation; *P* value of 0.05 was considered significant.

The study was approved by the local Ethical Committee of the University of Medical Sciences of Poznan.

## 3. Results

**Figure 4:**
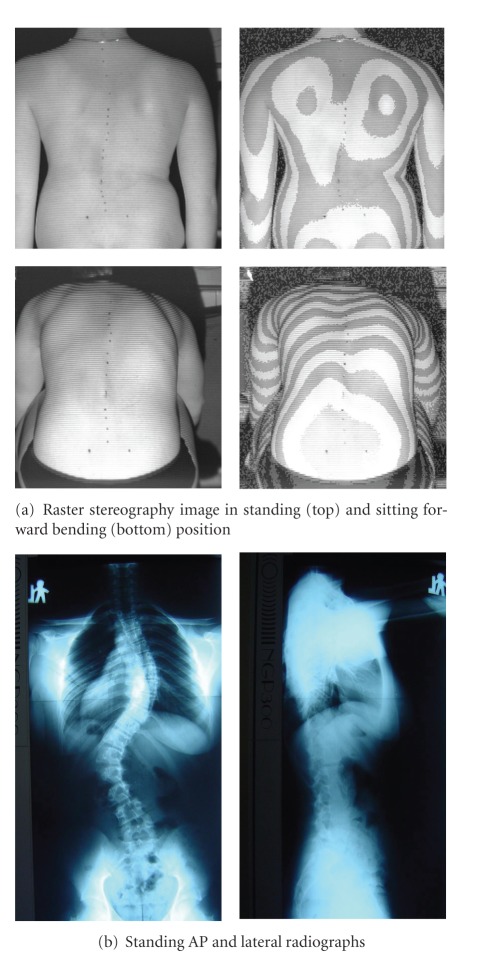
16 year old girl, treated with Cheneau brace for 1.5 years, including 5 months full time and 1.0 year part time wearing. Thoracic Cobb angle 54°, lumbar Cobb angle 60°, Risser sign 4. Two years after menarche. Main curve ATR = 10°, sum of three ATRs = 17°, POTSI = 16.0, HS = 14, (a) raster stereography; (b) radiographs.

**Figure 5:**
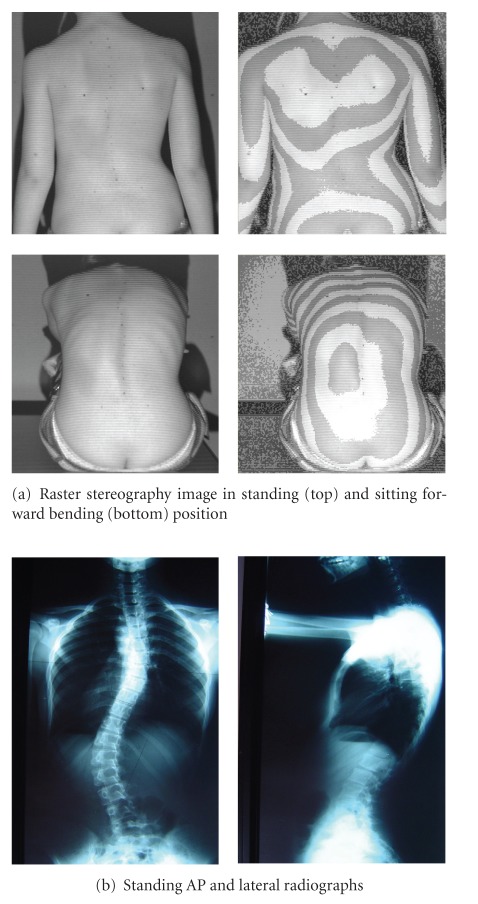
13 years and 9 months old girl, thoracic Cobb angle 26°, thoracolumbar Cobb angle 46°, Risser sign 4. 1.5 years after menarche. Main curve ATR = 15°, sum of three ATRs = 20°, POTSI = 50.2, HS = 18, (a) raster stereography, (b) radiographs.

In spite of similar Cobb angle the clinical parameters revealed discrepancy between the braced group (Figure 4), and the admitted for surgical treatment group (Figure 5), demonstrating less clinical deformity in the braced group, (Table 2).

Less clinical deformity in the braced group was found for the angle of trunk rotation (ATR main curve), which revealed significant differences between groups, *P* = 0.027, unpaired *t*-test with Welch correction. For the frontal plane assessment, neither the C7 plumb line (*P* = 0.83) nor the POTSI differ significantly between the groups (*P* = 0.19). The Hump Sum values were not quite significantly different (*P* = 0.07) as well as the axillary plumb line (*P* = 0.06).

There was no correlation between the primary curve Cobb angle and primary curve Bunnell angle in the braced group, *r* = 0.09 (*P* = 0.66 ns). There was a correlation between the primary curve Cobb angle and primary curve Bunnell angle in the admitted for surgical treatment group, *r* = 0.43 (*P* = 0.04).

## 4. Discussion

The aim of this study was to analyze discrepancy between surface image of the trunk and radiologically assessed curvature (Cobb angle) in adolescent girls undergoing treatment of progressive scoliosis with a brace versus nontreated girls admitted for surgical treatment and never treated before with corrective bracing. The Cobb angle at both groups achieved more than 45 degrees. The patients of the braced group refused surgical treatment to this time. The two groups were of the same age, with the same type of scoliosis and the Cobb angle.

 There was no significant difference in parameters describing frontal plane asymmetry, namely the POTSI index, C7 plumb line, and axillary plumb line. The expected difference between the two groups was in the amount of trunk rotation as measured clinically with the use of scoliometer We found that the rotational trunk deformity, evaluated with the scoliometer (ATR) and with surface topography (HS), was diminished in the braced group comparing to the group of girls nontreated with bracing. We noticed no significant correlation between ATR and Cobb angle in the braced group while we observed significant correlation between these parameters within the group admitted for surgical treatment. We conclude that wearing a corrective brace for more that one year was capable to change the trunk shape without influencing the Cobb angle.

 Discrepancy between surface image of the trunk and the radiological angle in idiopathic scoliosis was reported by Weiss [8]. In his study a girl with idiopathic scoliosis was observed for two years during brace treatment. The brace treatment improved clinical appearance in spite of an increase of Cobb angle. Goldberg et al. [9, 10], Grosso et al. [11], and Weiss [12] published on importance of comprehensive clinical assessment in scoliosis and underlined that clinical evaluation of the shape of the trunk in patients with idiopathic scoliosis cannot be substituted with radiological evaluation. On the other hand, the Brace Study Group of the Scoliosis Research Society published on criteria for adolescent idiopathic scoliosis brace studies and did not contain the parameters which describe the shape of the body of patients with idiopathic scoliosis. Clinical parameters describing scoliotic deformity were not included in the group of “potentially useful additional variables” [13]. However, other researchers claim that clinical deformity associated with adolescent idiopathic scoliosis should never be underestimated [14]. Watanabe et al. reported that most of the patients do not have negative self-image regarding back appearance when the thoracic curve angle is less than 30 degrees but have a negative self-image when the thoracic curve Cobb angle is more than 40 degrees and rotation angle is more than 20 degrees. Patients with greater Cobb angle or greater rotation angle at the thoracic curve had a negative self-image after surgery. Thus, thoracic scoliotic deformity with rib prominence should be substantially reduced by the surgical treatment to improve satisfaction and self-image [15].

The cosmetic appearance of the trunk in a patient with scoliosis does not depend solely on the magnitude of Cobb angle but on frontal trunk balance, thoracic hypokyphosis, frontal rib cage deformity, rib hump, waist asymmetry, and trunk rotation. An improved short-term effect on cosmetics using braces has been reported by other authors [16–19].

Finally, the discrepancy between the clinical and radiological parameters describing severe scoliosis after brace treatment which was observed in our study is an argument to systematically control the patients with radiography, as the scoliometer readings may be misleading if a corrective brace is regularly worn.

## 5. Conclusions

1. Adolescent girls with idiopathic scoliosis having the Cobb angle above 45 degrees and subjected to brace treatment for more than one year revealed smaller clinical deformity of their back comparing to nontreated girls having similar Cobb angle.
2. Both the parameters describing clinical deformity and the radiological angle should be considered in scoliosis evaluation due to possible discrepancy between the measurements.

## Figures and Tables

**Table 1:** Curve pattern in both groups.

Curve type	Lenke type	Braced group	Admitted for surgical treatment
Thoracic	I	13	13
Thoracolumbar	V	2	2
Double thor. and lumbar	III	8	7

All		23	22

**Table 2:** Values of the clinical parameters in braced patients versus admitted for surgical treatment patients matched for sex, age, and Cobb angle. The mean and standard deviation are presented. HS: Hump Sum. s: difference significant. ns: difference not significant.

Parameter	Brace-treated patients *n* = 23	Admitted for surgery patients *n* = 22	Significance of difference	*P* value
Cobb angle	55.0° ± 6.8°	59.7° ± 14.6°	ns	0.67
ATR main curve	11.9° ± 3.4°	15.1° ± 5.6°	s	0.027
ATR three levels	17.9° ± 4.7°	21.8° ± 6.1°	ns	0.2
HS standing	16.8° ± 3.8°	19.2° ± 4.6°	Not quite s	0.07
C7 plumb line	0.32 ± 1.7 cm	0.45 ± 1.9 cm	ns	0.83
Axillary plumb line	0.71 ± 2.1 cm	2.36 ± 3.1 cm	Not quite s	0.06
POTSI index	33.08 ± 18.3	41.36 ± 22.08	ns	0.19
